# The Medico-Literary Journal

**Published:** 1880-07

**Authors:** 


The Medico-Literary Journal, edited and published by Mrs. M. P. Sawtelle, M. D., of San Francisco, Cal., was mentioned some time ago among our exchanges; and it was our intention to allude to it again very soon thereafter; but procrastination, and probably the the time bestowed, not wasted, upon a bran new baby, especially, ought to plead some extenuation for our unintentional silence. Our better half insists that as we are not wanting in courtesy and politeness to her sex, the *amende honorable* should be made even now. We always try to treat our professional *brethren* in a kind and fraternal way; so we will have to beg our distant professional *sister* to put our negligence down to taking care of the recent introduction into the family, and if she has ever *experienced* such an advent into her domestic circle, "she knows how it is herself." The journal is full of well chosen articles and spicily written editorials on subjects of interest to both the profession and laity, and well deserves all the encouragement and patronage it receives.

